# Investigating Predictors of Visiting, Using, and Revisiting an Online Health-Communication Program: A Longitudinal Study

**DOI:** 10.2196/jmir.1345

**Published:** 2010-09-02

**Authors:** Jonathan Van 't Riet, Rik Crutzen, Hein De Vries

**Affiliations:** ^2^School for Public Health and Primary Care (CAPHRI)Maastricht UniversityMaastrichtNetherlands; ^1^Wageningen University and Research CentreAgricultural Economics Research InstituteThe HagueNetherlands

**Keywords:** Health communication, Web-based interventions, research subject selection, Internet, exposure

## Abstract

**Background:**

Online health communication has the potential to reach large audiences, with the additional advantages that it can be operational at all times and that the costs per visitor are low. Furthermore, research shows that Internet-delivered interventions can be effective in changing health behaviors. However, exposure to Internet-delivered health-communication programs is generally low. Research investigating predictors of exposure is needed to be able to effectively disseminate online interventions.

**Objective:**

In the present study, the authors used a longitudinal design with the aim of identifying demographic, psychological, and behavioral predictors of visiting, using, and revisiting an online program promoting physical activity in the general population.

**Methods:**

A webpage was created providing the public with information about health and healthy behavior. The website included a “physical activity check,” which consisted of a physical activity computer-tailoring expert system where visitors could check whether their physical activity levels were in line with recommendations. Visitors who consented to participate in the present study (n = 489) filled in a questionnaire that assessed demographics, mode of recruitment, current physical activity levels, and health motivation. Immediately after, participants received tailored feedback concerning their current physical activity levels and completed a questionnaire assessing affective and cognitive user experience, attitude toward being sufficiently physically active, and intention to be sufficiently physically active. Three months later, participants received an email inviting them once more to check whether their physical activity level had changed.

**Results:**

Analyses of visiting showed that more women (67.5%) than men (32.5%) visited the program. With regard to continued use, native Dutch participants (odds ratio [OR] = 2.81, 95% confidence interval [CI] = 1.16-6.81, *P* = .02) and participants with a strong motivation to be healthy (OR = 1.46, CI = 1.03-2.07, *P* = .03) were most likely to continue usage of the program. With regard to revisiting, older participants (OR = 1.04, CI = 1.01-1.06, *P* = .01) and highly educated participants (OR = 4.69, CI = 1.44-15.22, *P* = .01) were more likely to revisit the program after three months. In addition, positive affective user experience predicted revisiting (OR = 1.64, CI = 1.12-2.39, *P* = .01).

**Conclusions:**

The results suggest that online interventions could specifically target men, young people, immigrant groups, people with a low education, and people with a weak health motivation to increase exposure to these interventions. Furthermore, eliciting positive feelings in visitors may contribute to higher usage rates.

## Introduction

### Background

Cardiovascular diseases and cancers are the main causes of mortality in many Western countries. Because these and many other diseases are largely the result of unhealthy behaviors [1-3], an important goal of health communication is to encourage and motivate people to engage in healthful and disease-preventive behaviors. But how can we most effectively reach a large part of the population? In high-income countries, most adults now have access to the Internet. Online health communication thus has the potential to reach large audiences, with the additional advantages that it can be operational at all times and that the costs per visitor are low [4-7]. Furthermore, research shows that Internet-delivered interventions can be effective in changing health behaviors such as physical activity [8,9].The Internet thus seems to offer vast opportunities for health communication. However, the public health impact of interventions is only in part determined by reach and efficacy. According to the RE-AIM Model, which offers a framework for the evaluation of the public health impact of health promoting interventions [10,11], other important factors are adoption, implementation, and maintenance. In this model, adoption refers to the extent to which the intervention is integrated in relevant settings and maintenance refers to the extent to which the intervention is sustained over time. Implementation refers to the extent to which the intervention is delivered and used as intended. In the present study we focused on exposure, which is closely related to implementation [12]. Exposure can be construed as consisting of (1) visiting the intervention website, (2) continued use (ie, staying on the intervention website long enough to use and process the information), and (3) revisiting the intervention website [13]. Whereas exposure in the colloquial sense suggests passive reception of incoming information, we see exposure in an online context as entailing a certain degree of user control [13]. Investigating exposure to Internet-delivered interventions is important because evidence from efficacy trials indicates that exposure rates are generally low, which limits the potential public health impact of these interventions [14-16]. In some cases, people may have low motivation to access the online intervention [17,18]. In addition, previous research has established that both online research and online health-communication interventions suffer from high attrition rates [15,19,20]. In fact, several authors have argued that the ease with which people may disengage from online interventions might pose a major problem, both for online research and for online therapeutic applications or health-communication programs [15,21]. Instead of regarding high attrition rates as an inevitable reality of online health communication efforts or as merely a study limitation, however, it has been argued that researchers should build a “science of attrition” [15]. Insight into the determinants of visiting, using, and revisiting of online health-communication interventions could enable us to increase exposure to online health communication. For this reason, the present study investigated several potential determinants of exposure to an online health-communication program.

Previous research has identified several factors that can influence exposure to Internet-delivered health-communication interventions. First, intervention factors such as ease of enrollment and ease of dropout have been shown to influence exposure rates [15]. Personal factors, such as participants’ expectations before use and participants’ level of education [15], might also be important. According to Rogers [22], users with less formal education are more likely to discontinue the adoption of innovations. Therefore, people with a low education level may be expected to be less likely to use online health-communication programs. In addition, because women have been found to be more likely to use the Internet for searching health-related information than men [23,24], it could be expected that women might also be more likely to use online health-communication interventions. Previous research at our department has indeed shown that visitors to established Dutch Internet interventions were more highly educated and were more likely to be female than average among the Dutch population [25]. In addition, age might influence exposure to an online health-communication program, older people being more likely to use the Internet for information about health than adolescents [20,26]. In line with this, a recent study found that women, older people and people living in census tracts associated with higher socioeconomic status were more likely to enroll in an online dietary intervention trial [16]. A dearth of literature exists, however, on the impact of other potentially important determinants of exposure to Internet-delivered health-communication interventions. One such factor might be participants’ motivation to pursue and maintain health [27]. Participants who are motivated to live a healthy lifestyle might be more inclined to visit, use and revisit online health-communication programs. Another potentially important factor is user experience, which refers to what a person thinks and feels during and after a visit [28]. A positive user experience during and after the first visit might be a prerequisite for staying long enough to complete the intervention and for revisiting.

### Objectives

In the present study, we aimed to investigate demographic, behavioral and psychological determinants of exposure to an online program promoting physical activity. We assessed demographic variables, participants’ current physical activity level and their motivation to pursue and maintain health [27]. To investigate the role of user experience [28], we also assessed participants’ reactions toward the online content. Because previous research has shown that affective and cognitive beliefs are separate entities and can influence decision making differently (eg, [29,30]), we investigated both affective and cognitive components of user experience. We also assessed attitudes toward being sufficiently physically active and intentions to be sufficiently physically active. Attitudes and intentions have been shown to be closely related to actual health behavior [31] and have also been shown to be related to increased interest in health-promoting interventions in general [32,33] and online health-promoting interventions specifically [13]. In the present study, we investigated whether attitudes and intentions with regard to physical activity could lead to increased use of online activity-promoting interventions.

In sum, the Internet offers vast possibilities for health-communication efforts. Unfortunately, online health-communication interventions are characterized by low exposure rates. The present study sought to investigate predictors of visiting, using, and revisiting online interventions to increase our knowledge of exposure to these interventions.

## Method

### Recruitment

**Figure 1:**
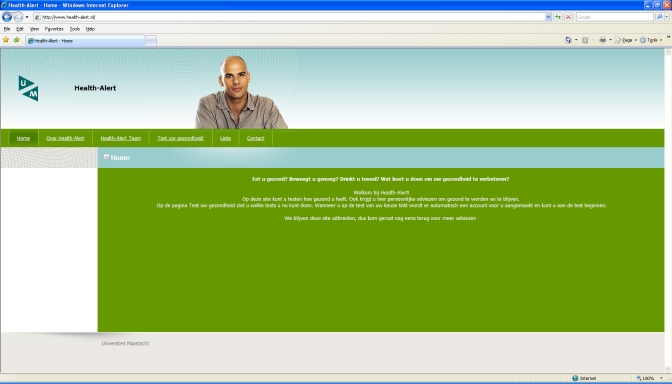
Homepage of the Health-Alert website

A webpage was created providing the public with information about health and healthy behaviour (www.health-alert.nl). (See Figure 1 for an impression of the visual impact of the website.) To recruit participants, we aimed to generate publicity for the website through local media. Two news items were broadcasted on local television and several articles appeared in local newspapers in the province of Limburg in The Netherlands. In addition, we approached webmasters of related Dutch websites (ie, websites about exercise, physical activity, weight control, or other health- or activity-related subjects) and asked them to link to our website. In total, we approached 16 webmasters, 7 of whom obliged. On our Health-Alert webpage, we created a hyperlink called “physical activity check” that led to a physical activity computer-tailoring expert system where visitors could check whether their physical activity levels were in line with recommendations. Dutch recommendations with regard to physical activity state that healthy adults should be physically active for at least thirty minutes on at least five days of the week [34]. Visitors were told that they would receive an invitation to again check their physical activity levels three months from the first assessment so that they could check whether their physical activity levels had changed in this period. Before entering the questionnaire, visitors were asked whether they consented to their answers being used for scientific research or whether they wanted to use the intervention but not participate in the research. Participants were also told that entering their email address was necessary for them to participate in the research. The minimum age required for participation was 18 years; participants younger than 18 could also use the intervention, but only adults were eligible to participate in the study. The majority of eligible participants (489/593 or 82.5%) consented to being included in the research.

### Design and Procedure

**Figure 2:**
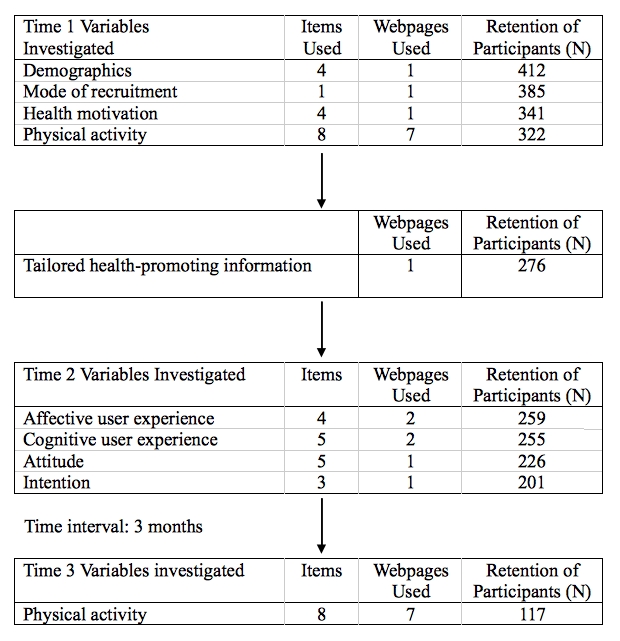
Flowchart of the study

The present study used an observational longitudinal design. After giving informed consent to participate, participants were invited to complete a questionnaire that assessed participants’ demographics, mode of recruitment, health motivation, and current physical activity levels (Time 1). Immediately after, participants were provided with a short message about the Dutch recommendations for physical activity. After participants were informed about these recommendations, they received tailored feedback concerning their current physical activity level. This tailored feedback informed them whether or not they met the recommendations for physical activity and offered tips on how to increase their physical activity or maintain their (already sufficient) current level of physical activity. The feedback was tailored solely to current physical activity levels and was not tailored to demographic or psychosocial variables. After reading the tailored feedback, participants completed an additional questionnaire assessing affective and cognitive user experience, attitude, and intention (Time 2). Three months later, participants received an email inviting them to participate in an assessment of whether their physical activity level had changed (Time 3). Figure 2 depicts a flowchart of the study, showing the assessed constructs, the number of items that were used for each construct, the number of webpages that were used for each construct, and the number of participants that completed all measures for each construct. Completion of the study at Time 1 and Time 2 took 30 minutes on average, whereas completion of the Time 3 measures took only 5 minutes on average. All 489 participants who entered the study were invited to complete the three-month follow-up questionnaire. In case of nonresponse, participants were sent one reminder email one week later. The study was conducted from November 2007 through March 2008 and was approved by the Medical Ethical Committee of Maastricht University.

### Questionnaire at Time 1

#### Demographics and Mode of Recruitment

We assessed gender, age, ethnicity (1 = native Dutch; 2 = nonnative Dutch) and education (1 = low education; 2 = medium education; 3 = high education). In the complex schooling system in the Netherlands, a low education level refers to primary or basic vocational school, a medium education level refers to secondary vocational school or high school, and a high education level refers to advanced vocational school or university. In addition, we asked participants to indicate how they learned about the physical activity check (1 = through a search engine, eg, Google; 2 = through a link on another website; 3 = through a newspaper; 4 = through family, friends or co-workers; 5 = through local television).

#### Health Motivation

To measure participants’ health motivation, a 4-item questionnaire was used (based on [27]). Participants indicated their agreement with the following items on a 7-point scale (1 = I do not agree at all to 7 = I totally agree): “I often think about making sure that I am healthy.” “I often think about staying healthy,” “In general, I aim to prevent poor health,” and “I often think about preventing poor health.” These items were averaged to create a general health motivation score (Cronbach alpha = .80). In a pilot study among 171 university students aimed at testing the health motivation scale, it was found to have acceptable test-retest stability over a one-week period (*r* = .65) and to have small to medium-sized correlations with a number of health behaviors such as vegetable and fruit consumption and exercise.

#### Assessment of Baseline Physical Activity

Physical activity levels were assessed using the short version of the International Physical Activity Questionnaire (IPAQ) [35]. Two questions were asked to assess vigorous physical activity, The first question asked participants to indicate on how many days during the past week they had engaged in vigorous physical activity (eg, exercising). The second question assessed the time participants typically spend being vigorously physically active on such a day (in minutes). The two measures were multiplied to arrive at a total score of vigorous physical activity per week. In addition, two questions were asked to assess moderate physical activity. The first of these asked participants to indicate on how many days during the past week they had engaged in moderate physical activity (eg, gardening or cycling). The second assessed the time participants typically spend being moderately physically active on such a day (in minutes). The two measures were multiplied to arrive at a total score of moderate physical activity per week. Finally, vigorous and moderate physical activity were added up to arrive at a total score of physical activity during the past week and divided by seven to obtain a score of physical activity per day [35]. Because the Dutch recommendations for physical activity do not include walking [34], the walking-items of the IPAQ were not used to calculate the total physical activity score. We found that scores for 4 respondents indicated they had been physically active for more than eight hours a day on all days of the week, a physical activity level that we judged highly unlikely. Thus, we truncated the physical activity variable to a maximum of eight hours a day. Not truncating the physical activity variable or omitting these cases from the analyses yielded similar results and identical conclusions.

### Questionnaire at Time 2

#### Affective User Experience

Two items assessed positive affective reactions to the online content (ie, positive affective user experience); these items assessed the extent to which participants thought the content made them feel happy (1 = very happy to 7 = not at all happy) and relieved (1 = very relieved to 7 = not at all relieved). Scores were reversed and combined to create an average score. (Cronbach alpha = .75). Two items assessed negative affective reactions to the online content (ie, negative affective user experience) and assessing the extent to which participants thought the content made them feel sad (1 = very sad to 7 = not at all sad) and afraid (1 = very afraid to 7 = not at all afraid). Scores were reversed and combined to create an average score (Cronbach alpha = .83).

#### Cognitive User Experience

Five items assessed cognitive user experience by asking participants to indicate the extent to which they thought the online content was relevant (1 = very relevant to 7 = not at all relevant), interesting (1 = very interesting to 7 = not at all interesting), objective (1 = very objective to 7 = not at all objective), and exaggerated (1 = very exaggerated to 7 = not at all exaggerated). Furthermore, one item asked participants to indicate the extent to which participants agreed with the content (1 = I totally agree to 7 = I totally disagree). After we reversed the scores of all items except the exaggerated item, the scores on the five items were averaged (Cronbach alpha = .75).

#### Attitude

Five items were used to assess attitude toward physical activity asking participants to indicate on semantic differentials the extent to which they rated engaging in at least thirty minutes of physical activity for at least five days of the week as: 1 = very good to 7 = very bad; 1 = very important to 7 = very unimportant; 1 = very sensible to 7 = not sensible at all; 1 = very nice to 7 = not at all nice; 1 = a lot of fun to 7 = no fun at all. After scores on the attitude items were reversed an average score was created (Cronbach alpha = .90).

#### Intention

Three items were used to assess intention to be physically active. Two items asked participants to indicate whether they planned to be physically active for at least thirty minutes a day on at least five days of the week and whether they considered being physically active for at least thirty minutes a day on at least five days of the week (1 = definitely not to 7 = definitely). One item asked participants: “How likely is it that you will be physically active for at least thirty minutes a day on at least five days of the week in the coming six months?” (1 = very unlikely to 7 = very likely). An average intention score was calculated (Cronbach alpha = .89).

### Questionnaire at Time 3: Physical Activity

During the three-month follow-up, physical activity levels were assessed using the same procedure as in the pretest questionnaire (ie, using the IPAQ).

### Outcome Measures

The present study had three main outcome measures: visiting the website at Time 1, using the website at Time 1, and revisiting the website at Time 3. Determinants of visiting were investigated by comparing the demographics of the study sample with those of the general Dutch population. To assess continued use, we recorded which webpages were accessed by participants. A dichotomous variable was created that indicated whether participants had continued in the program up to the point of being exposed to the tailored health-promoting information (0 = dropped out before exposure to the information; 1 = continued use up to exposure to the information). Revisiting at Time 3 was assessed by means of a dichotomous variable indicating participation at Time 3 (0 = did not revisit at Time 3; 1 = did revisit at Time 3).

### Statistical Analysis

First, we investigated the demographic profile of the sample and the prevalence of the different modes of recruitment by means of descriptive analyses. Second, logistic regression analyses were performed to investigate whether continued use of the study could be predicted by gender, age, ethnicity, education (we created two dummy variables to be able to estimate the contribution of the three education groups), mode of recruitment (we created four dummy variables to be able to estimate the contribution of the five modes of recruitment groups), health motivation, and baseline physical activity. Third, logistic regression analyses were performed to investigate which variables could predict participation at Time 3. In step 1 of the logistic regression analyses, the Time 1 variables were entered (demographic variables, mode of recruitment, health motivation, physical activity), and in step 2, the Time 2 variables were entered in addition (affective user experience, cognitive user experience, attitude and intention). We used the statistical package SPSS 15.0 (SPSS Inc, Chicago, IL, USA) for the analyses. To calculate the statistical power of this study to reject false null hypotheses, we conducted a post-hoc statistical power test [36,37]. With 16 predictors in the regression analysis, an observed R^2^ of 0.22 (see Table 5), a sample size of 489 and alpha = .05, the test results indicated an observed power of 1.0.

## Results

### Participants

In total, 489 people participated in the study. The sample consisted of more women (n = 336; 67.5%) than men (n = 162; 32.5%). Age ranged from 18 to 86 years, with a mean age of 38.6 years (SD 15.0). Reflecting the general Dutch population, most of the participants were native Dutch. Approximately one-third of participants (n = 186) had a high education level, 48.6% (n = 242) had a medium education level, and 14.1% (n = 70) had a low education level. In the general population these percentages are 29.0%, 42.3%, and 28.6% respectively [38].

**Table 1:** Demographic profile of participants

Variable		Percentage
**Sex**		
	Male	32.5
	Female	67.5
**Ethnicity**		
	Native	81.5
	Nonnative	18.5
**Education**		
	Low	14.1
	Medium	48.6
	High	37.3
**Recruitment**		
	Search engine	28.6
	Hyperlink on related website	48.7
	Advertisement in newspaper	3.5
	Through family, friends, coworkers	2.2
	Local television	17.0
**Physical activity (minutes/day)**		
	0-15	21.3
	16-30	16.9
	31-45	15.7
	46-60	12.0
	61 or more	34.1

### Recruitment

The majority of participants indicated that they had learned about the physical activity check on related websites. Percentages for mode of recruitment, as well as additional demographics and physical activity levels, are presented in Table 1.

### Analyses of Continued Use

Of the 489 participants who enrolled in the study, 276 (55.4%) continued in the study up to the point of being exposed to the tailored information. We conducted a logistic regression analysis to investigate whether demographics, mode of recruitment, or physical activity levels could predict continued use at the first measurement. Results of the logistic regression analyses showed that native Dutch participants completed more pages than nonnative participants. Furthermore, participants with a strong health motivation completed more pages than those with a weak health motivation (Table 2). Combined, all variables accounted for 8% of the variance in continued use (Nagelkerke R^2^ = .08).

**Table 2:** Results of the logistic regression analysis with continued use as the dependent variable

	Odds Ratio (OR)	95% Confidence Interval (CI)	Wald χ^2^	*P* Value^a^
Gender	1.22	0.67-2.39	0.51	.48
Age	1.02	1.00-1.04	2.29	.13
Medium education^b^	0.97	0.40-2.35	0.01	.94
High education^b^	1.44	0.56-3.66	0.57	.45
Ethnicity (0 = nonnative Dutch; 1 = native Dutch)	*2.81*	*1.16-6.81*	*5.23*	*.02*
Recruitment through other website^c^	1.43	0.73-2.83	1.08	.30
Recruitment through newspaper^c^	0.79	0.14-4.33	0.07	.79
Recruitment through family, friends, coworkers^c^	1.03	0.18-5.75	0.00	.98
Recruitment through television^c^	0.92	0.40-2.13	0.04	.85
Health motivation	*1.46*	*1.03-2.07*	*4.51*	*.03*
Physical activity	1.00	1.00-1.00	0.02	.88

^a^ Significant effects (*P* < .05) are indicated by italics.

^b^ “Low education” as reference group

^c^ “Search engine” as reference group

### Analyses of Revisiting at Time 3

Of all 489 participants, 126 (25.3%) participated in the three-month follow-up assessment (Time 3), of which 117 (23.5%) completed all measures. We first conducted correlation analyses to investigate whether revisiting was associated with physical activity, health motivation, positive affective user experience, negative affective user experience, cognitive user experience, attitude, intention, and continued use. (We used Spearman’s ρ as a measure of all correlations involving continued use and revisiting and Pearson’s *r* for all other correlations.) These analyses showed that revisiting was positively correlated with health motivation, positive affect, cognitive user experience, and continued use, and negatively with negative affect (see Table 3).

Furthermore, we conducted a logistic regression analysis to investigate which variables could predict revisiting at Time 3. Results of step 1 of the logistic regression analyses showed that older participants were more likely to participate at Time 3. In addition, highly educated participants were more likely to participate than participants with a low education level (Table 4). Nagelkerke R^2^ for this analysis was .18. In step 2 of the analyses (Table 5), age and education were still significant predictors of participation at Time 3, but in addition, positive affective user experience contributed significantly to the prediction of revisiting at Time 3. The more participants indicated that the computer-tailored feedback had made them feel good, the more likely they were to participate in the study at Time 3. Negative affective user experience, cognitive user experience, attitude, and intention did not have significant effects on revisiting at Time 3. Nagelkerke R^2^ for this analysis was .22.

**Table 3:** Correlations, means, and standard deviations for continued use, revisiting, and other variables

Variables^a^		1	2	3	4	5	6	7	8	Mean	SD	
(1) Physical activity										68.71	85.06	
(2) Health motivation		.04								5.96	0.77	
	*P* value	.49										
(3) Positive affect		*.22*	*.23*							4.40	1.09	
	*P* value	< .01	< .01									
(4) Negative affect		-.10	-.07	*-.39*						2.43	1.34	
	*P* value	.10	.23	< .01								
(5) Cognitive user experience		.03	.10	*.36*	*-.16*					5.18	0.88	
	*P* value	.61	.14	< .01	.02							
(6) Attitude		*.21*	*.37*	*.32*	*-.16*	*.26*				5.92	0.85	
	*P* value	< .01	< .01	< .01	.01	< .01						
(7) Intention		*.27*	*.21*	*.27*	*-.25*	*.15*	*.67*			5.49	1.34	
	*P* value	< .01	< .01	< .01	< .01	.02	< .01					
(8) Continued use		.00	.04	-	-	-	-	-		0.55	0.50	
	*P* value	.97	.43									
(9) Revisiting		.08	*.10*	*.18*	*-.18*	*.13*	.03	.09	*.19*	0.25	0.44	
	*P* value	.13	.04	< .01	< .01	.049	.60	.16	< .01			

^a^ Significant correlations (*P* < .05) are indicated by italics.

**Table 4:** Results of step 1 of the logistic regression analysis with participation at Time 3 as the dependent variable

Variables^a^	OR	95% CI	Wald χ^2^	*P* Value
Gender	1.03	0.58-1.85	0.01	.91
Age	*1.05*	*1.03-1.07*	*18.64*	*.00*
Medium education^b^	2.15	0.85-5.44	2.61	.11
High education^b^	*3.54*	*1.39-9.00*	*7.03*	*.01*
Ethnicity (0 = nonnative Dutch; 1 = native Dutch)	1.14	0.58-2.21	0.14	.71
Recruitment through other website^c^	1.33	0.69-2.59	0.72	.40
Recruitment through newspaper^c^	1.36	0.38-4.93	0.22	.64
Recruitment through family, friends, coworkers^c^	1.37	0.23-8.27	0.12	.73
Recruitment through television^c^	2.09	0.95-4.62	3.34	.07
Health motivation	1.24	0.86-1.77	1.33	.25
Physical activity	1.00	1.00-1.01	0.98	.32

^a^ Significant effects (*P* < .05) are indicated by italics.

^b^ “Low education” as reference group

^c^ “Search engine” as reference group

**Table 5:** Results of step 2 of the logistic regression analysis with participation at Time 3 as the dependent variable

Variables^a^	OR	95% CI	Wald χ^2^	*P* Value
Gender	0.90	0.44-1.83	0.09	.77
Age	*1.04*	*1.01-1.06*	*7.99*	*.01*
Medium education^b^	2.84	0.92-8.78	3.28	.07
High education^b^	*4.69*	*1.44-15.22*	*6.61*	*.01*
Ethnicity (0 = non-native Dutch; 1 = native Dutch)	0.96	0.45-2.05	0.01	.92
Recruitment through other website^c^	1.07	0.46-2.48	0.03	.87
Recruitment through newspaper^c^	1.66	0.34-8.22	0.39	.53
Recruitment through family, friends, co-workers^c^	1.44	0.19-11.20	0.12	.73
Recruitment through television^c^	1.49	0.55-4.00	0.62	.43
Health motivation	1.13	0.69-1.85	0.22	.64
Physical activity	1.00	1.00-1.01	0.25	.62
Positive affective user experience	*1.64*	*1.12-2.39*	*6.56*	*.01*
Negative affective user experience	0.88	0.67-1.15	0.91	.34
Cognitive user experience	1.06	0.71-1.59	0.09	.77
Attitude	0.90	0.54-1.52	0.15	.70
Intention	0.97	0.70-1.34	0.03	.86

^a^ Significant effects (*P* < .05) are indicated by italics.

^b^ “Low education” as reference group

^c^ “Other” as reference group

## Discussion

The aim of the present study was to identify demographic, psychological and behavioral determinants of exposure to an online health-communication program advocating physical activity.

### Visiting

The results concerning visiting the website revealed that most participants were women and that, in comparison to the total Dutch population, our sample was highly educated. Almost half of our participants were recruited through links on related websites, suggesting that for online health-communication interventions, the Internet can be a valuable place for recruitment but that additional methods may be needed to attract more men and lower educated adults.

### Using

First, with regard to continued use, our results showed that native Dutch participants completed more pages than nonnative participants. It is unclear why nonnative visitors were less likely to use the program than native Dutch visitors. More research is needed to identify the needs of specific ethnic populations and the potential reasons for limited usage of health-promoting programs in this group. Ethnic targeting (see for instance [39]) may be a powerful tool to increase exposure rates in specific ethnic populations.

Second, participants who were highly motivated to live a healthy lifestyle were more likely to use the program. This suggests that when people are sufficiently motivated to live a healthy lifestyle, they will be more likely to search the Internet for specific health-related information such as computer-tailored advice. To reach individuals with a weak health motivation, it is conceivable that other types of content that relates to the interests of these individuals but does not necessarily relate to health may be used to attract this target group. If participants’ attention can be attracted with non health-related content, this might make it easier to engage people who are not intrinsically motivated to live a healthy lifestyle. Social marketing strategies may contribute to the development of appealing health-communication websites because, in marketing, much effort is expended to understand the needs of target groups and to create an exchange in which these needs can be fulfilled [40,41].

### Revisiting

Our analyses of revisiting at Time 3 showed that age predicted participation at the three-month follow-up: older participants were more likely to participate at Time 3. These results suggest that it can be especially difficult to obtain high exposure rates when targeting online health-promoting programs at young people. Since young people mainly use the Internet for getting in touch with friends and potential friends and chatting [42], research is needed to explore the potential of health-communication programs that aim to make participation more attractive by including possibilities for social interaction. Furthermore, highly educated participants were more likely to participate at Time 3 than participants with a low education. Future research should investigate how we can design online interventions that are interesting for people with a low education level. Here as well, social marketing principles [40,41] may be helpful.

Our last finding might offer an additional answer to this question. We found that participants were more likely to revisit the program when an earlier visit had resulted in positive feelings whereas there was no significant effect of cognitive user experience on revisiting. This underlines the importance of user experience [28] but suggests that affective experiences might be more important than cognitive experiences. It is also in line with the assertion that an important attraction of the Internet constitutes its vast possibilities for “gratification” [43]. The next question is which elements in the online content produced high levels of positive affective user experience. Future research could investigate which strategies are most effective in increasing positive affect and whether increasing positive affect can result in increased exposure. Positive framing of health-related information might be one way of achieving this [44,45]. Future studies should investigate whether information framing or other techniques, such as cartoons or clips, can be helpful in increasing exposure by eliciting positive feelings. Perhaps this can be of particular use to attract people with a low education level.

### Strengths and Limitations

A strength of the present study is the fact that we obtained a sample of participants from the general population and observed them in a real-life health-communication context, which contributes greatly to the ecological validity of our findings. Furthermore, our main outcome measures, visiting, using, and revisiting, did not depend on self-report measures but were objectively assessed.

One limitation of our study was the fact that our sample was predominantly female and modest in size. A more representative and larger sample could have contributed to greater validity and could have provided us with more certainty with regard to whether the results can be generalized. However, the fact that the online intervention investigated in the study attracted more women than men can in itself be an interesting result, suggesting that women are more interested in online health-promoting programs than men (see [46] for similar results). Also, a power calculation showed that the study had adequate power to detect significant effects.

A second limitation of our study was the fact that the intervention that we used was relatively simple, consisting of questionnaires and tailored advice at two points in time. Future studies could offer visitors a much wider range of possibilities, varying from watching videos to participating in chat-boxes. By objectively tracking visitors in such interventions, researchers can obtain more sophisticated information on the determinants of exposure.

A further issue concerns our investigation of continued use. Several questions from the Time 1 questionnaire (ie, demographic variables, mode of recruitment, and health motivation) were included solely for the sake of the study and were not used in the tailored feedback. It could be argued that a lack of interest in answering these questions could have caused participants to drop out of the study. This would then not be an accurate reflection of poor continued use of a health website. Future studies could employ shorter questionnaires or limit questionnaires to contain only questions that are relevant to the tailored feedback. We note, however, that for any tailored intervention, it is essential that visitors continue the program long enough to be able to finish the necessary questions. Even though in the present study not all questions were used for the tailored feedback, we argue that our measure of continued use served as a useful proxy for continued use of online tailored health-promoting programs.

Another potential limitation may have been the fact that a reminder was sent to participants after three months. It is unclear whether participants revisited the intervention because they remembered the intervention or because of the reminder. The reminder email thus constituted a confounding factor. Yet, many online health-promoting programs make use of prompts or reminders by email [47]. It is therefore of interest to investigate predictors of revisiting under such conditions. Future studies could employ an experimental design, in which reminders are sent to only half of the participants, to investigate whether predictors of revisiting are similar when participants receive a reminder versus when no reminder is sent.

A final limitation was the fact that we used an observational design. Research using experimental manipulations aimed to influence exposure rates can offer stronger grounds for the causality of the effects. In a recent study, for instance, Albarracín and colleagues [48] experimentally tested the effects of procedures aiming to increase exposure, called “meta-interventions” by the authors. They found that when the intervention was introduced to potential participants in a way that left them some degree of choice as to whether participants would want to change their health-related behavior, the intervention attracted more participants than when the intervention was introduced as highly effective in changing behavior. Future research should further explore the effectiveness of such meta-interventions. Based on the results of the present study, these interventions could be targeted specifically at men, immigrant groups, young people, people with a low education level, and people with a weak health motivation.

### Conclusion

The Internet offers vast possibilities for health-communication efforts. Unfortunately, online health-communication interventions are characterized by low exposure rates. The present study sought to investigate predictors of visiting, using, and revisiting to increase our knowledge of exposure to online interventions. The results showed that women were more likely to visit the website than were men. Furthermore, native Dutch participants and participants with a strong health motivation were most likely to continue usage of the website. Older participants, highly educated participants, and participants who reported high levels of positive affective user experience were most likely to revisit the website. Online health-communication interventions could be specifically targeted at men, young people, immigrants, and people with low education levels. Engaging non health-related content might be used to attract the attention of those people who are not intrinsically motivated to live a healthy lifestyle. Furthermore, it is important that interventions offer participants sufficient opportunities for enjoyment.

## Abbreviations

IPAQ: International Physical Activity Questionnaire
